# Association between maternal cannabis use and birth outcomes: an observational study

**DOI:** 10.1186/s12884-020-03371-3

**Published:** 2020-12-11

**Authors:** Camilla A. Michalski, Rayjean J. Hung, Ryan A. Seeto, Cindy-Lee Dennis, Jennifer D. Brooks, Joanna Henderson, Robert Levitan, Stephen J. Lye, Stephen G. Matthews, Julia A. Knight

**Affiliations:** 1 Dalla Lana School of Public Health, University of Toronto, Toronto, Ontario Canada; 2 Lunenfeld-Tanenbaum Research Institute, Sinai Health System, Toronto, Ontario Canada; 3 Lawrence S Bloomberg Faculty of Nursing, University of Toronto, Toronto, Ontario Canada; 4 St. Michael’s Hospital, Toronto, Ontario Canada; 5 Centre for Addiction and Mental Health, Toronto, Ontario Canada; 6 Department of Psychiatry, University of Toronto, Toronto, Ontario Canada; 7 Department of Physiology, University of Toronto, Toronto, Ontario Canada; 8 Department of Obstetrics and Gynecology, University of Toronto, Toronto, Ontario Canada; 9 Department of Medicine, University of Toronto, Toronto, Ontario Canada

**Keywords:** Cannabis, Marijuana, Epidemiology, Canada, Women, Pregnancy, Small for gestational age, Birth weight

## Abstract

**Background:**

As cannabis consumption is increasing globally, including among pregnant women, there is a critical need to understand the effects of cannabis on fetal development and birth outcomes. We had two objectives: to determine 1) the factors associated with self-reported cannabis use in the pre/early-pregnancy period, and 2) whether cannabis use is associated with low birth weight, preterm birth, or small size for gestational age (GA) infants.

**Methods:**

Maternal questionnaire and birth outcome data was gathered from 2229 women and 1778 singleton infants in the Ontario Birth Study, a hospital-based prospective cohort study (2013–2019). Women self-reported cannabis use within 3 months of learning their pregnancy status. Multivariable linear and logistic regression was conducted to 1) identify factors associated with cannabis use, and 2) determine the associations between cannabis use with the selected birth outcomes.

**Results:**

Cannabis use increased in the cohort over time. Women who reported cannabis use (*N* = 216) were more likely to be younger and more likely to use alcohol, tobacco, and prescription pain medication, although most did not. These women had infants born at lower average birth weights and had 2.0 times the odds of being small for GA (95% confidence interval: 1.3, 3.3) after multivariable adjustment for socioeconomic factors and other substance use.

**Conclusion:**

Our results suggest that women who use cannabis around the time of conception have higher odds of having infants that are small for gestational age. Targeted clinical messaging may be most applicable to women actively trying to conceive.

**Supplementary Information:**

**Supplementary information** accompanies this paper at 10.1186/s12884-020-03371-3.

## Background

Cannabis use is rising globally, and has more than doubled among Canadians between 1985 and 2015 [1]. It is also cited as the most commonly used illicit substance during pregnancy [2, 3]. Studies have shown cannabis use to be highest in early first trimester, followed by substantial drop-off as the pregnancy progresses [4, 5]. As consumption gains social acceptance, and in light of evidence that suggests endocannabinoid involvement in early reproductive events [6], pregnant women and their children stand to benefit from targeted research concerning birth outcome effects of prenatal cannabis use.

While some existing studies have already presented a link between maternal cannabis use and adverse birth outcomes such as low birth weight and preterm birth, others continue to report no association [7, 8]. One of the biggest challenges in this research area lies in isolating the independent association between cannabis use and birth outcomes, given that many cannabis users use alcohol and tobacco concurrently [3].

As such, a homogeneous study population with low rates of concurrent substance use could better isolate the association in question. Utilizing such a study population, our research endeavour had two main objectives: first, to determine which factors are associated with maternal cannabis use in the pre/early-pregnancy period, and second, to analyze the association between cannabis use during this period with the following birth outcomes: low birthweight, preterm birth, and small size for gestational age (GA).

## Methods

### OBS study design

The study was conducted using data from the Ontario Birth Study (OBS), an ongoing prospective pregnancy and birth cohort study established at Mount Sinai Hospital, Toronto, Canada. Eligible participants included women 18 years of age or older within their first or early second trimester of pregnancy (≤ 17 weeks GA). Between January 2013 and June 2019, 6950 women were approached for recruitment at antenatal clinics at Mount Sinai, of which 2973 (43%) consented to participate. Twenty-four subsequently withdrew, leaving 2949 women in the current cohort.

In the OBS, the collection of biological samples, lifestyle questionnaires (LSQs), and clinical data is integrated with routine clinical care. LSQs are collected at three time points and can be completed electronically or on paper. The first, LSQ1, is usually administered between 12 and 16 weeks of gestation, LSQ2 between 24 and 32 weeks of gestation, and LSQ3 between 6 and 10 weeks postpartum**.** Additional information concerning the study cohort can be found elsewhere [9]. The OBS has been approved by Mount Sinai’s Research Ethics Board and all participants have provided informed written consent (REB #11–0321-E).

### Analytic datasets

**Fig. 1:**
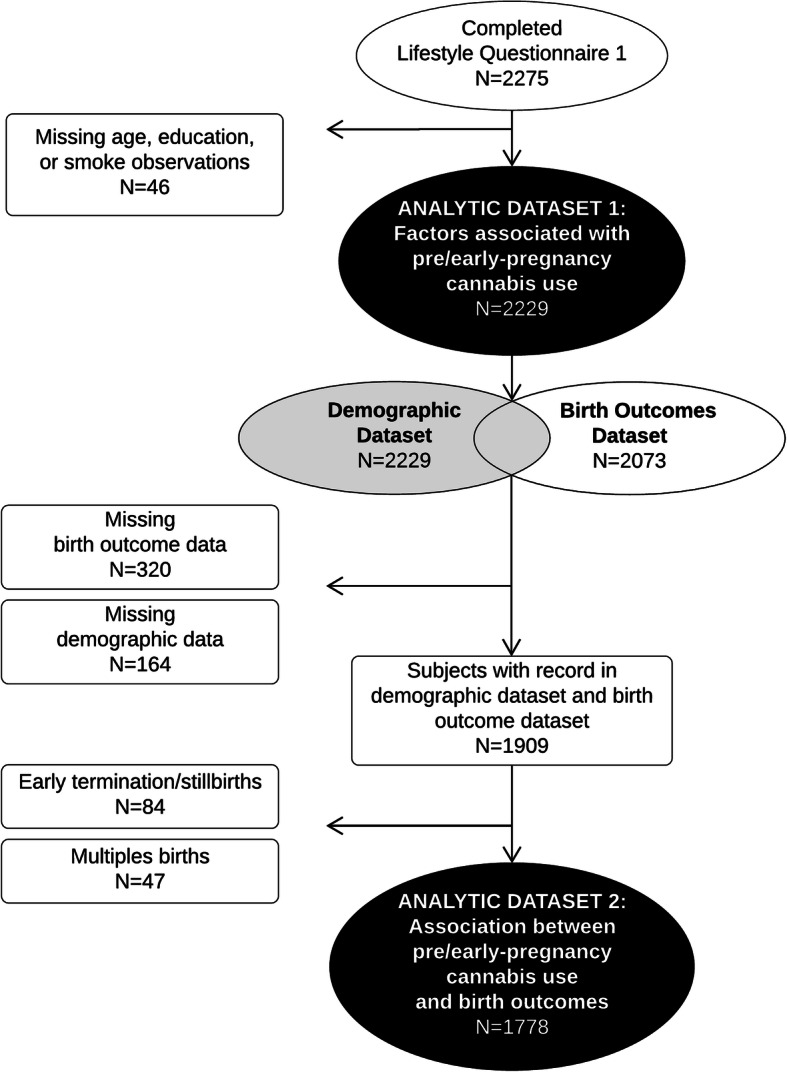
Flowchart of exclusion criteria applied to create analytic datasets

Of the 2949 women in the cohort, LSQ1 was completed and entered into the database for 2275 women at the time of data extraction (June 2019). Maternal age was missing for six participants and they were excluded. Observations with missing education (*N* = 19) or tobacco smoking measures (*N* = 21) were also excluded because they produced empty cells when cross-tabulated with pre/early-pregnancy cannabis use (Fig. 1). The final sample available for this analysis was 2229 women.

For the birth outcome models, this dataset (*N* = 2229) was then merged with birth outcome hospital records (*N* = 2073). Of these, 1778 observations were singleton, live births with corresponding maternal LSQ1 data; 5 observations were missing gestational age, and 3 were missing birthweight which were excluded from the corresponding regression analyses.

### Cannabis use

Pre-pregnancy cannabis use was identified in LSQ1 with the following prompt, *“In the 3 months before you knew you were pregnant, did you use any of the following drugs on your own without a doctor’s prescription?”* If “*marijuana or hashish*” was one of the options selected, then the participant was designated a pre/early-pregnancy cannabis user. If not selected, the participant was designated a non-cannabis user. One woman did not select “*marijuana or hashish*” for this question but reported using Nabilone (a synthetic cannabinoid) as a medication within 3 months of learning she was pregnant, and she was included as a pre/early-pregnancy cannabis user. This participant did not report continued use during pregnancy. No other participants reported using cannabis as a medication.

### Birth outcomes

Pregnancy outcome information was derived from clinical data collected from hospital records. Stillbirth and twin pregnancies were excluded from analyses of birth outcomes. Separate regression models were created based on four outcome measures: birth weight (*continuous*), low birth weight *(low/not low),* preterm birth *(preterm/term),* and small size for GA *(small/not small).*

Low birth weight was defined as a birth weight less than 2500 g [10]. Preterm birth was defined as a live birth before 37 weeks of pregnancy. Small size for GA was defined as having a sex- and GA- specific birth weight less than the 10th percentile of the most recently published Canadian population-based reference group [11].

### Covariates

Covariates were chosen for model inclusion based on factors identified in the literature as being associated with either cannabis use or fetal development and birth outcomes. All multivariable regression models included age, year of LSQ1 completion*,* pre-pregnancy body mass index (BMI), household income, education, ethnicity, alcohol use, tobacco use, anxiety or depression symptoms, prescription anti-depressant use, and prescription pain medication use. Notably, smaller ethnicity categories had to be collapsed into coarser groups due to issues with convergence. Birth outcome models also adjusted for infant sex and GA.

Depression symptoms were measured using the 2-item Patient Health Questionnaire (PHQ-2), and anxiety symptoms were measured using the 2-item Generalized Anxiety Disorder scale (GAD-2). A score greater than or equal to 3 on either PHQ-2 or GAD-2 was used to define depression and/or anxiety for the covariate measure. Meta-analyses of validation studies suggested a sensitivity of 0.89 (95% CI 0.81–0.95) and specificity of 0.76 (95% CI 0.70–0.81) for PHQ-2, and 0.76 (95% CI 0.55–0.89) and 0.81 (95% CI 0.60–0.92) for GAD-2 [12, 13].

Prescription antidepressant and pain medication use was identified through the following prompts, “*In the past six months, have you taken any prescription medicines? Please include only medicines that have been prescribed by your doctor*.” If “*depression/anxiety medications*” or “*pain medications*” were selected, then the participant was considered a user of the respective medication.

### Statistical analyses

Descriptive summary statistics were calculated for each variable. To determine a trend in cannabis use over time, a Cochran-Armitage test was conducted. In total, five outcomes were analyzed: pre/early-pregnancy cannabis use (*yes/no)* for the first study objective*,* and birth weight *(continuous),* low birth weight *(low/not low),* preterm birth (*preterm/term),* and small size for GA *(small/not small)* for the second study objective*.* Linear regression was used for the continuous birth weight outcome, and the average birth weight of infants born to cannabis-using mothers (compared to non-cannabis-using mothers, in grams) was reported. Logistic regression was used for binary outcomes, and odds ratios (ORs) were reported.

Missing observations were kept in the model and only dropped if they produced an empty cell when cross-tabulated with the outcome of interest. For pre-pregnancy BMI (*N* = 122) and household income (*N* = 147), the median was imputed for the missing values. The multivariable regression models were run with robust cluster analysis, such that if an individual participated in the OBS for more than one pregnancy, these pregnancies were clustered together (*N* = 53).

To further rule out the potential effects of other substance use, three sensitivity analyses were conducted: [1] participants reporting smoking tobacco during pregnancy were excluded, [2] participants reporting consuming any alcohol during pregnancy were excluded, and [3] participants reporting either smoking tobacco or consuming alcohol during pregnancy were excluded. For [2] and [3], additional analyses also excluded observations with missing alcohol consumption information.

Data processing and analyses were performed using Stata version 14.0 (Stata Corporation, College Station, TX).

## Results

### Study population

Among the 2229 women included for analysis of factors associated with pre/early-pregnancy cannabis use, the mean age (standard deviation) was 33.7(3.8) years at baseline, and pre-pregnancy BMI was 23.4(4.6). The majority (74.2%) of the cohort reported a household income of more than $100,000 per year, with 41.1% reporting a graduate degree. About half of the cohort reported non-Jewish European ethnicity (53.8%). Jewish ethnicity was the third most commonly reported at 14.9% (many women with Jewish heritage seek care at Mount Sinai Hospital due to historical ties to the Jewish community).

With respect to cannabis measures, 216 (9.7%) women reported use in the three months before knowing they were pregnant. On average, women reported finding out they were pregnant 4.30(±1.30) weeks into gestation.

With respect to birth outcomes, 51.2% of infants were born male. Low-weight births and preterm births had a prevalence of 4.8% (*N* = 85) and 5.9% (*N* = 105), respectively. Small for GA births were more common with a prevalence of 9.6% (*N* = 170). Additional demographic factors and their distributions are reported in Table 1.

**Table 1:**
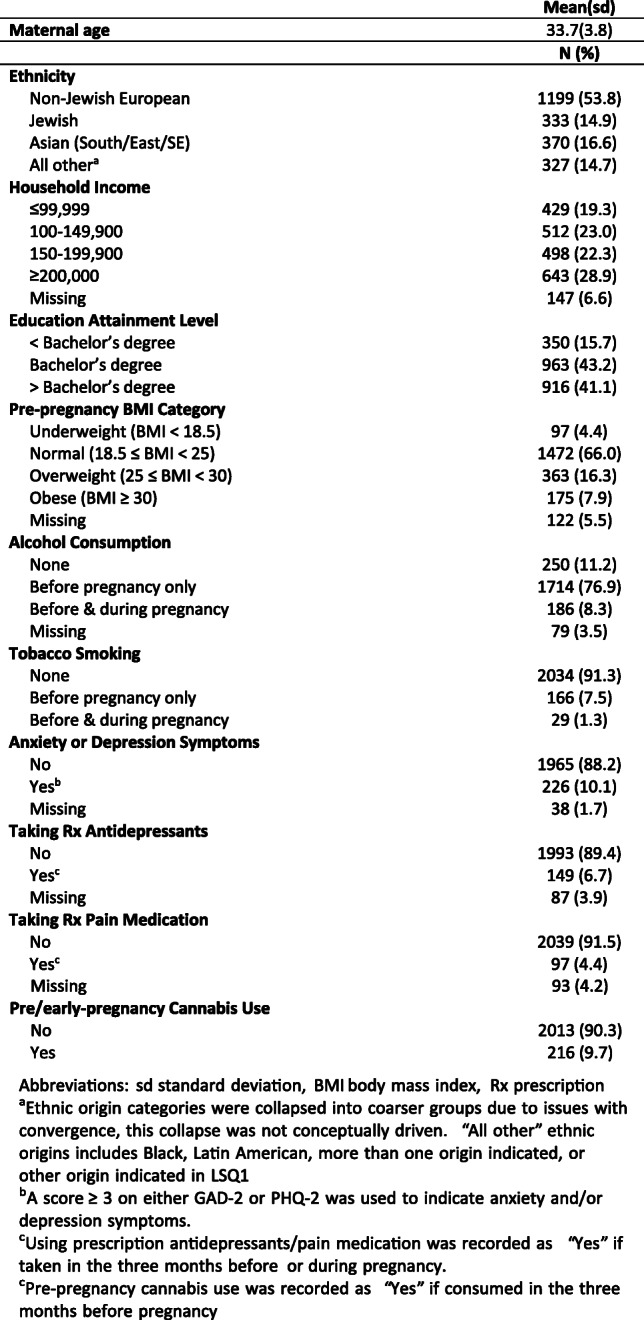
Demographic characteristics of the Ontario Birth Study maternal population (N = 2229, 2013–2019)

### Factors associated with pre/early-pregnancy cannabis use

**Fig. 2:**
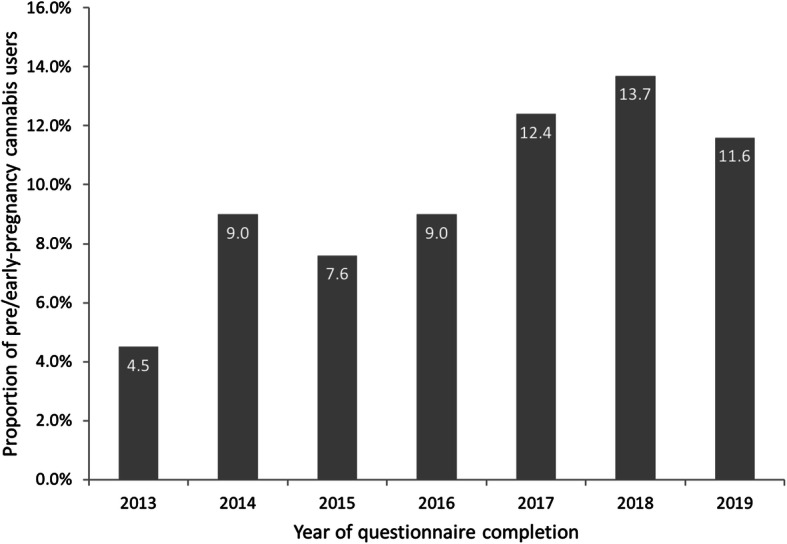
Proportion of study participants reporting pre/early-pregnancy cannabis use, per year (2013-2019)

Women had lower odds of reporting cannabis use in the pre/early-pregnancy period if they were older (OR: 0.92, 95% CI: 0.89, 0.97 for every additional year of age), and higher odds if they had completed the questionnaire in more recent years (OR: 1.19, 95% CI: 1.10, 1.29 per calendar year) (Table 2). A Cochran-Armitage test analyzing changes in prevalence of use over time also suggested a significant 1.4% increase in self-reported pre/early-pregnancy cannabis use per year (Fig. 2). Women reporting Jewish ethnicity had marginally higher odds of reporting cannabis use compared to those reporting non-Jewish European ethnicity (OR: 1.50, 95% CI: 0.99, 2.26), whereas women reporting Asian ethnicities had comparatively lower odds (OR: 0.46, 95% CI: 0.25, 0.87). Compared to the lowest household income level (≤$99,999), those that reported the highest income (≥ $200,000) exhibited 0.47 times the odds of reporting pre/early-pregnancy cannabis use (95% CI: 0.30, 0.80).

**Table 2:**
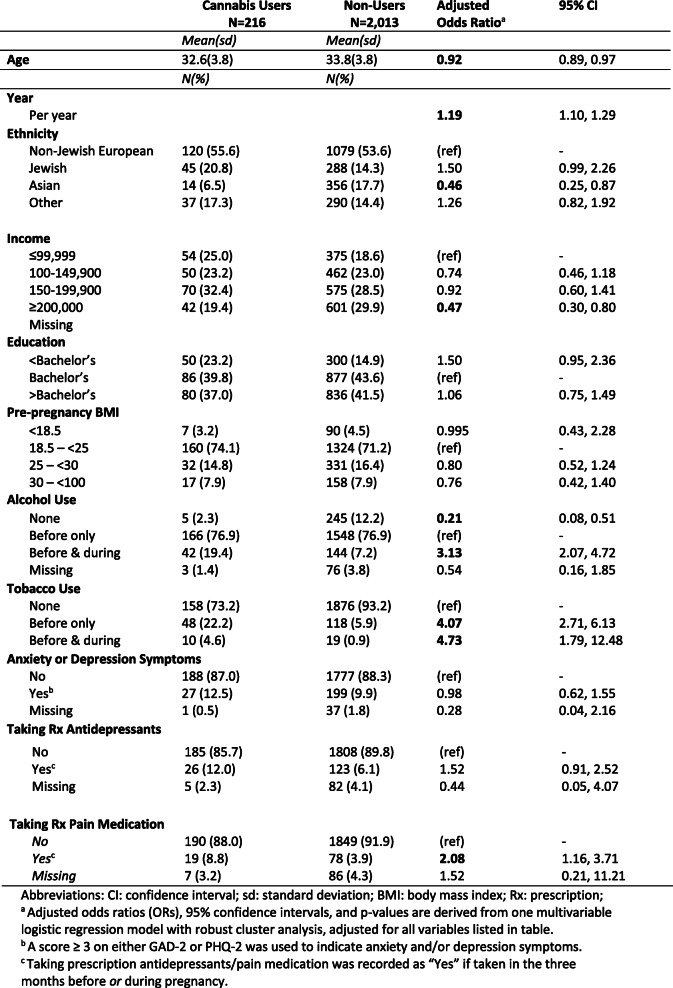
Odds ratios for factors associated with pre-pregnancy cannabis use (N= 2229, 2013-2019)

Women who reported not having consumed any alcohol in the year before pregnancy had 0.21 times the odds of reporting cannabis use (95% CI: 0.08, 0.51), whereas those that reported consuming alcohol both before and during pregnancy exhibited 3.13 times the odds of reporting pre/early-pregnancy cannabis use (95% CI: 2.07, 4.72) compared to women who consumed alcohol only before pregnancy (the majority of women). Compared to non-smokers, women who smoked tobacco before pregnancy only, and those that smoked tobacco during pregnancy exhibited higher odds of pre/early-pregnancy cannabis use than non-smokers (OR: 4.07, 95% CI: 2.71, 6.13; and OR: 4.73, 95% CI: 1.79, 12.48, respectively). Taking prescription pain medication was also associated with pre/early-pregnancy cannabis use (OR: 2.08, 95% CI: 1.16, 3.71). Pre-pregnancy BMI, education level, anxiety and/or depression symptoms, and antidepressant use were not associated with pre/early-pregnancy cannabis use in this population.

### Association between cannabis use and birth outcomes

Infants born to mothers reporting pre/early-pregnancy cannabis use weighed 86g less on average than those born to mothers reporting no pre/early-pregnancy cannabis use (95% CI: − 155, − 17) (Table 3). No association was found with low birth weight as a binary measure, nor preterm birth. Offspring of women who reported pre/early-pregnancy cannabis use had 2.03 times the odds of being small for GA (95% CI: 1.25, 3.31) (Table 3).

**Table 3:** Effect measures of maternal cannabis use on selected birth outcomes

Outcome	Coefficient	95% CI
**Birth weight** ^a^ *(grams)*		
Non-Users	(ref)	
Users	**−85.8**	−**154.6, −17.2**
	**Odds Ratio**	**95% CI**
**Low birth weight** ^a^		
Non-Users	(ref)	
Users	0.93	0.29, 2.93
**Preterm birth** ^b^		
Non-Users	(ref)	
Users	1.26	0.62, 2.57
**Small size for GA** ^b^		
Non-Users	(ref)	
Users	**2.03**	**1.25, 3.31**

*N* = 1773 for preterm birth model, *N* = 1770 for birth weight and small size for GA models

Abbreviations: *CI* confidence interval, *GA* gestational age

^a^Continuous (linear) and binary (logistic) birth weight regression models with robust cluster analysis adjusted for infant sex, GA (continuous), pre/early-pregnancy cannabis use, year of LSQ1 completion, maternal age, ethnicity, education, income, pre-pregnancy BMI, alcohol use, tobacco use, anxiety/depression symptoms, antidepressant use, and pain medication use.

^b^Preterm birth and small for GA logistic regression models adjusted for infant sex, pre/early -pregnancy cannabis use, year of LSQ1 completion, maternal age, ethnicity, education, income, pre-pregnancy BMI, alcohol use, tobacco use, anxiety/depression symptoms, antidepressant use, and pain medication use

Sensitivity analyses with differing population exclusions based on tobacco and alcohol consumption during pregnancy suggested that the magnitude and direction of the associations remained generally consistent. In short, the magnitude and direction of the findings stayed consistent after the removal of these observations. In all analyses, women who consumed cannabis had small for GA infants, ranging from an odds ratio of 1.83 to 2.14. Women who consumed cannabis also generally had lower birth weight infants, ranging from 64.8 to 98.3g lower, although some results (i.e. analyses where current smokers were removed) became marginally statistically insignificant (see Supplementary Table 1).

## Discussion

We found that pre/early-pregnancy cannabis use increased in our study conducted before and after the legalization of recreational use in Canada. In this study, which followed over 2000 women, we found that those who used cannabis were younger and were more likely to drink alcohol, smoke tobacco, and use prescription pain medication. Reported use was lower in women in the highest income category but did not differ across other income categories. With respect to birth outcomes, we found that women who reported cannabis use in the 3 months prior to learning they were pregnant had infants born at lower average birth weights, and these infants had higher odds of being small for GA.

With respect to factors associated with cannabis use, our findings fall in line with those in comparable studies. For instance, Corsi and colleagues (2019) examined population-based data in Ontario over a similar time period and found that pregnant women who use cannabis are younger than their non-using counterparts and are more likely to drink alcohol and smoke tobacco [14].

The associations found between maternal cannabis use and birth outcomes are also consistent with other comparable studies. Corsi and colleagues (again examining population-based data in Ontario), similarly found that cannabis use reported at some point during obstetrical care is associated with infants born small for GA (as well as pre-term birth, placental abruption, and transfer to neonatal intensive care) [15].

Another Canadian study conducted in British Columbia also reported that women who reported using cannabis during their first prenatal visit had increased odds of having a small for GA infant, spontaneous preterm birth, and intrapartum stillbirth [16].

In terms of biological plausibility, previous literature has suggested that endocannabinoids can cross the placental barrier [17, 18]. Moreover, cannabinoid receptors and their endogenous ligands have been detected in the earliest stages of embryonic development, with the ECS appearing to play essential roles in these early stages for neuronal development and cell survival [6, 19, 20].Taken together, there exists biological plausibility that in-utero cannabis exposure may cause fetal growth abnormalities and influence birth outcomes. In fact, recent evidence has suggested that the ECS may play a role in placentation; altered placental ECS expression has been associated with spontaneous miscarriage [21]. Additional studies have also proposed that cannabis may affect glucose and insulin regulation, which could also influence fetal growth [22].

Our study extends the findings from existing literature through the homogeneity of our study population, which lends the advantage of better handling residual confounding. Even among our relatively healthy, high SES population exhibiting low rates of concurrent substance use, associations with adverse birth outcomes were found. Not only did the vast majority of the study population report high education attainment and household income, but the single centre hospital-based population effectively minimized confounding effects related to differences in quality of care. Notably, though the homogeneity of the population can be a major advantage, it also limits the generalizability of the study findings. For instance, 84.3% of women in our study had attained at least a Bachelor’s degree, compared to 50.4% across the Canadian population [23].

Additionally, all our covariate measures were captured on an individual basis and provided a high level of detail. Compared to similar studies using population-based registries, we did not estimate socioeconomic status, but instead had direct self-reports of income and education. We also captured detailed information on concurrent substance use including alcohol, tobacco, pain medication, and antidepressants. The data collection method likely provided more reliable covariate measures, especially concerning substance use. The aforementioned comparable studies collected these measures through antenatal care providers, wherein a patient may be more influenced by social desirability bias when asked to disclose substance use. Because OBS questionnaires are self-administered and not reported to a health care provider, we may have better minimized the risk of misclassification. The prevalence of pre/early-pregnancy use in our cohort (9.7%) was similar to the prevalence of use (10.0%) in women aged 25 to 44 years in Ontario in the 2012 Canadian Community Health Survey [24].

In terms of illicit substance use, only 50 women (< 5%) in our study reported use in the 3 months before pregnancy, which was not associated with cannabis use (*p* = 0.63). General smoking rates were also lower among our cannabis-using population compared to other studies (73.2% non-smokers among our cannabis users versus 29.7% among the Corsi et al. provincially representative population) [14]. These low rates of concurrent use helped further isolate the association in question. Our additional sensitivity analyses further strengthened our argument that concurrent tobacco use cannot explain all of the observed associations with cannabis.

Lastly, our study captured an early time point of cannabis use. As mentioned, previous studies have suggested that self-reported cannabis use is highest in the early first trimester, followed by substantial drop off as the pregnancy progresses [﻿5﻿]. Thus, our pre/early-pregnancy measure questioning participants about their cannabis use 3 months before learning their pregnancy status may be capturing a crucial window of high cannabis exposure, which other studies overlooked (of the 1778 women in the birth outcome models, only 10 reported using cannabis during pregnancy, 1510 reported no use, and 258 were missing a measure). It is possible that the associations we observed with the pre/early-pregnancy use period are due to under-reported use during pregnancy; the proportion of women reporting use during pregnancy in our study is lower than that reported in other studies [25].

There are a number of potential limitations that should be considered. Because our cannabis use measure was self-reported, its prevalence may be understated as some women who use cannabis may be misclassified as non-users. Non-disclosure rates may especially affect observations that were collected before recreational cannabis use was legalized, when stigma was higher. This reduction in stigma surrounding use may also contribute to our finding that cannabis use increased over time. However, this misclassification would bias our findings towards the null, suggesting that the true association may actually be larger than the reported results. Urinalysis screenings were not available, although it is important to consider that no gold standard measure exists. Differing rates of excretion, metabolism, cannabinoid potency, and half-life times affect urinalysis accuracy [26]. In fact, El Marroun and colleagues (2011) showed substantial agreement between urinalyses and self-reported measures [26].

Our measure of cannabis use also did not take frequency, amount, or mode of delivery into account. While deeper insight into women’s patterns of use could prove fruitful and help distinguish between light and heavy users, the variability in individual tolerance and cannabinoid concentrations poses significant barriers to the applicability of such measures. Regarding potential differences between different modes of consumption, data from the National Cannabis Study (2019) suggests that compared to men, Canadian women are much more likely to report any other mode of consumption other than smoking compared to men [27]. Deeper insights into the potential differences in risk between these different modes are warranted in light of growing trends in alternative modes of consumption, such as edibles and vaping.

## Conclusion

We found that pre-pregnancy (before women knew that they were pregnant, which likely includes early pregnancy) cannabis use is associated with lower mean birth weight and increased odds of having an infant born small for GA. Targeted clinical messaging may be most applicable to women who are actively trying to conceive.

## Supplementary Information

**Additional file 1: Supplementary Table 1:** Effect measures of maternal cannabis use on select birth outcomes with different population exclusion criteria applied.

## Data Availability

The participant data used to support the findings of this study were from the Ontario Birth Study and are not freely available to respect the confidentiality of the participants, ensure data integrity, and avoid scientific overlap between projects. Approval for use of the data requires a research proposal subject to review by the Ontario Birth Study Steering Committee, as well as approval by institutional research ethics boards. Additional details are available on the study website at www.ontariobirthstudy.com

## Abbreviations

BMI: Body mass index
CI: Confidence interval
GA: Gestational age
GAD-2: 2-item Generalized Anxiety Disorder scale
LSQ: Lifestyle questionnaire
OBS: Ontario Birth Study
OR: Odds ratio
PHQ-2: 2-item Patient Health Questionnaire
SD: Standard deviation
