# Supplementary material for: Association between maternal cannabis use and birth outcomes: an observational study
Source: BMC Pregnancy Childbirth. 2020 Dec 11;20:771. doi: 10.1186/s12884-020-03371-3 (PMC7731469; doi:10.1186/s12884-020-03371-3)
Supplement: Supplementary file 1 — Additional file 1: Supplementary Table 1: Effect measures of maternal cannabis use on select birth outcomes with different population exclusion criteria applied. [file 12884_2020_3371_MOESM1_ESM.docx]

Supplementary Table 1: Effect measures of maternal cannabis use on select birth outcomes with different population exclusion criteria applied.

| *Model* | *N removed* | **Coeff. (95%CI)** | **Odds Ratio (95% CI)** | | | | |
| --- | --- | --- | --- | --- | --- | --- | --- |
|  |  | **Birth weight (g)** | **Low birth weight** | | **Preterm birth** | **Small size for GA** | |
| Original fully-adjusted model |  | **-85.8**  **(-154.6, -17.2)** | 0.93  (0.29, 2.93) | 1.26  (0.62, 2.57) | | | **2.03**  **(1.25, 3.31)** |
| Remove  current smokers | *19* | -64.8  (-133.4, 3.8) | 0.74  (0.21, 2.64) | 1.16  (0.55, 2.42) | | | **1.83**  **(1.90, 3.06)** |
| Remove  current drinkers | *136* | **-93.04**  **(-168.4, -17.7)** | 1.38  (0.43, 4.45) | 1.16  (0.55, 2.42) | | | **2.07**  **(1.24, 3.43)** |
| Remove  current/missing drinkers | *206* | **-98.30**  **(-174.9, -21.7)** | 1.53  (0.46, 5.02) | 1.18  (0.54, 2.55) | | | **2.14**  **(1.29, 3.60)** |
| Remove  current smokers & current drinkers | *150* | -73.87  (-149.0, 1.3) | 1.13  (0.31, 4.09) | 1.04  (0.46, 2.35) | | | **1.89**  **(1.11, 3.22)** |
| Remove  current smokers & current/missing drinkers | *219* | **-79.63**  **(-156.1, -3.1)** | 1.29  (0.35, 4.71) | 1.07  (0.47, 2.43) | | | **1.97**  **(1.15, 3.36)** |
